# The Effects of Pre-Fermentative Addition of Oenological Tannins on Wine Components and Sensorial Qualities of Red Wine

**DOI:** 10.3390/molecules21111445

**Published:** 2016-10-31

**Authors:** Kai Chen, Carlos Escott, Iris Loira, Juan Manuel del Fresno, Antonio Morata, Wendu Tesfaye, Fernando Calderon, Santiago Benito, Jose Antonio Suárez-Lepe

**Affiliations:** Chemistry and Food Technologies Department, Polytechnic University of Madrid, Avenida Complutense S/N, 28040 Madrid, Spain; chenkai96@foxmail.com (K.C.); c.escott@alumnos.upm.es (C.E.); iris.loira@upm.es (I.L.); juan.fresno.florez@alumnos.upm.es (J.M.d.F.); antonio.morata@upm.es (A.M.); wendu.tesfaye@upm.es (W.T.); fernando.calderon@upm.es (F.C.); joseantonio.suarez.lepe@upm.es (J.A.S.-L.)

**Keywords:** oenological tannins, anthocyanins, wine colour, volatile compounds, sensorial properties, cluster analysis, principal component analysis

## Abstract

Today in the wine industry, oenological tannins are widely used to improve wine quality and prevent oxidation in wine aging. With the development of tannin products, new oenological tannins are developed with many specific functions, such as modifying antioxidant effect, colour stabilization and aroma modifications. The aim of this work is to investigate effects of pre-fermentative addition of oenological tannins on wine colour, anthocyanins, volatile compounds and sensorial properties. In this case, Syrah juice was extracted with classic flash thermovinification from fresh must in order to release more colour and tannins. Three types of oenological tannins, which are, respectively, derived from grape skin, seed (*Vitis vinifera*) and French oak (*Quercus robur* and *Querrus petraea*), were selected to carry out the experiments with seven treatments. Results indicated that tannin treatments significantly improved wine aroma complexity and sensorial properties. However, the concentration of some stable pigments such as Vitisin A, Vitisin A-Ac and Vitisin B was negatively affected by tannin additions. Nevertheless, by means of cluster analysis and principal component analysis, it was observed that higher alcohols were significantly promoted by grape seed tannin while most anthocyanins can be improved by addition of grape tannins. In conclusion, low amount of oenological tannin derived from grape seed is a promising method to be applied especially for young red wine making.

## 1. Introduction

It is well known that oenological tannins and other phenolic compounds potentially influence wine components and contribute to the sensorial properties during red winemaking [1,2,3]. Tannins play an important role during the aging process, modify aromatic complexity, prevent colour loss, create a balanced proportion of anthocyanins and improve wine structure [3,4,5]. Appropriate pre-fermentative addition of oenological tannin can positively contribute to colour stabilization and aroma protection during red wine aging [6,7]. Tannins can be divided into two groups, condensed tannins (so-called proanthocyanidins) of grapes origin; and hydrolysable tannins present in oak. Hydrolysable tannins contain large amounts of ellagitannins which can only be extracted from oak [1,8].

Depending on different fermentation scenario and grape varieties, single implement or hybrid application of oenological tannins can improve wine quality and correct must flaws [9]. Hydrolysable tannins originating from oak possess higher antioxidant activity than some tannins extracted from grape skin or seed [10,11]. However, experienced winemakers would prefer to use grape tannins rather than oak tannins mainly due to a significant modification of aroma complexity and colour stability from grape tannin [3,6]. More importantly, application dosage should be carefully considered, because over-adding exogenous oenological tannins may result in a dramatic decrease of total phenolic concentrations after alcoholic fermentation and negatively affect mouth feel and wine structure [3,4]. Nevertheless, adding oenological tannin to a young red wine containing a small amount of anthocyanin-acetaldehyde-flavonoid polymers can facilitate violet shift after alcoholic fermentation (AF) [12,13].

On the other hand, the most important purpose using oenological tannins is to improve wine colour stability, which can influence consumer perception. The colour diversity of wine is mainly due to the presence of different grape anthocyanins or monomeric anthocyanins, which are located in grape skins [14,15,16]. However, these anthocyanins are unstable and potentially polymerize to form stable pigments [17]. In addition, some of them can be degraded by oxidation. Thus, the anthocyanin profile of a wine may vary depending on many factors such as the grape variety and ripeness, winemaking techniques, yeasts used and aging time [18,19]. Based on recent research, the oenological tannins (derived from grape seed and skin) containing pyranoanthocyanin and flavanol-anthocyanin were commercially developed and widely applied to improve the colour stability of young red wine due to their highly resistance to oxidation and sulphur dioxide bleaching effect [8,9]. Additionally, the change of polarity of must can result in wine colour loss since anthocyanin solubility is reduced with an increase in ethanol content by yeast metabolism [17]. Moreover, the polymerization of anthocyanin is also correlated with precipitation of colouring matter during alcoholic fermentation (AF) [18,19]. However, further research should be done for analysing the specific effects of different oenological tannins on colour contributions or volatile compounds.

The addition of oenological tannins during red wine making could influence colour performance, volatile compounds, pigmentation and sensorial properties [3,6,8,10,20,21]. In this work, three typical oenological tannins, which were extracted from grape skin, seed and oak, respectively, were utilized to study the specific influence on wine colour, aroma and tasting characters for Syrah must fermentation.

## 2. Results and Discussion

### 2.1. The Influence of Oenological Tannins on Phenolic Parameters

As a practical tool, colour intensity and hue have been used for wine colour evaluation in previous studies [4,16]. Based on the results from Table 1, hue in Tanicol Vintage (TV) treatments was comparatively lower than other treatments while the hue of control was the highest among these seven treatments. Additionally, control had the lowest colour intensity of all the treatments. With the increase of tannin addition, the colour intensity in each tannin treatment was significantly increased because of the colour improvement by introduction of these tannins which contain some pigments originated from grapes and oak [9,22]. The gelatine index highlights the capacity of wine tannins to react with salivary proteins, which is responsible for the sensation of astringency experienced when tasting red wine [23]. The Robletan Estructura (RE) treatments with a low amount of tannin addition showed to be more astringent than other treatments, except for Tan Sutil (TS) 40 mg/L. PVPP has the same effect as Gelatine. Based on the results of Table 1, PVPP index can be increased by the addition of oenological tannin. HCL index reflects the state of polymerization of tannins in red wines [8], tannin treatment showed high values compared with the control, and the degree of tannin polymerization of TV is positively correlated with addition volume, opposite to the effects of RE and TS. In addition, vanillin index, which was indicated as the concentration of catechin, represented low-molecular weight flavonoids which react with vanillin and produce a red coloured product with absorbance at 500 mm [22]. As a result, both oak tannin RE and grape skin tannin TS are significantly contributed to vanillin index of red wine after AF while grape seed tannin is less effective than them. Ethanol index indicated the percentage of tannins combined with polysaccharides [24]; Control had the highest percentage of tannin that bond with the polysaccharides in all seven treatments. Ethanol index was impaired by the increase of tannin addition, though its level was not distinct among TV and TS treatments. TS treatment showed a positive relation with increasing tannin dosage. Finally, it is understandable that both total tannin and anthocyanin concentration were significantly raised by introduction of oenological tannins which may contain some of the grape anthocyanins or resulting from copigmentation during AF.

### 2.2. The Influence of Oenological Tannins on CIELAB Scales

**Figure 1:**
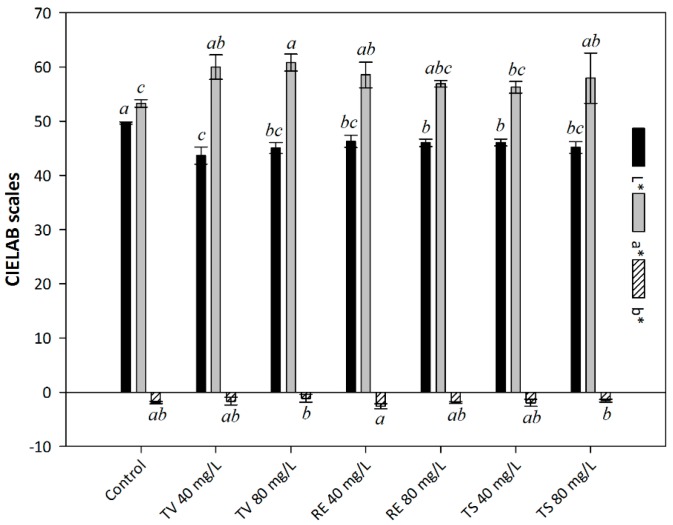
The influence of oenological tannins on CIELAB scales, L* (dark or light), a* (red or green) and b* (yellow or blue). The following nomenclature is used: assays fermented with Tanicol Vintage (TV) 40 and 80 mg/L, Robletan Estructura (RE) 40 and 80 mg/L as well as Tan Sutil (TS) 40 and 80 mg/L. Different letters indicate the significant differences were conducted by Duncan´s new multiple range test at *p* < 0.05.

CIELAB scales can easily lead to a better understanding of colour values. For the results in Figure 1, every L* scale was close to 50, indicating this batch of Syrah wines presented dark colour. L* scale of control was higher than that in tannin-treated trials. Meanwhile, a* scale is partly correlated with monomeric anthocyanins, tannin-treated trials showed more red colour, around 8% higher than control. The addition of RE was showing a negative relation on a* scale of Syrah wine. This tendency was observed on L* scale as well. Nevertheless, all these wines, especially for the addition of RE 40 mg/L, were presenting blue instead of yellow according to negative b* scale, which is related to wine age as well as the amount of monomeric anthocyanins [21,25].

### 2.3. The Influence of Oenological Tannins on Wine Anthocyanins

**Figure 2:**
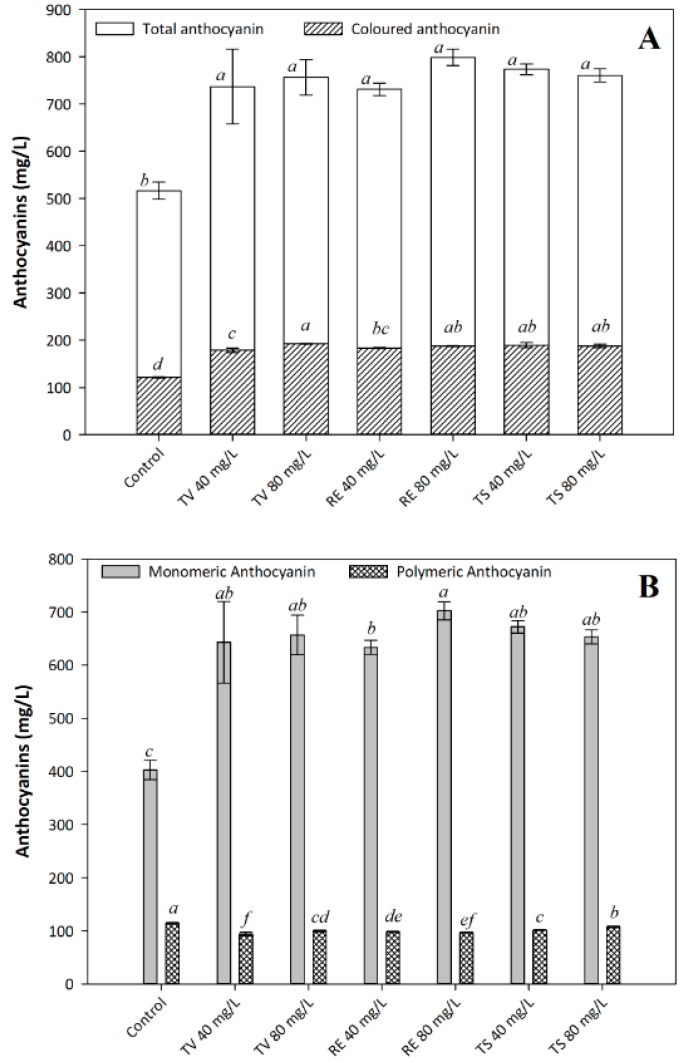
The concentration of total and coloured anthocyanin (**A**); and the concentration of polymerized anthocyanin and monomeric anthocyanin (**B**). The following nomenclature is used: assays fermented with Tanicol Vintage (TV) 40 and 80 mg/L, Robletan Estructura (RE) 40 and 80 mg/L as well as Tan Sutil (TS) 40 and 80 mg/L. Different letters indicate the significant differences were conducted by Duncan´s new multiple range test at *p* < 0.05.

Wine anthocyanins are mainly extracted from the aqueous phase during maceration and responsible for most of the colour performance in red wine [15]. Exogenous tannin additives can positively influence wine anthocyanin profiles with antioxidant effects and colour stabilization [22]. The colour of the wine is mainly due to the presence of monomeric anthocyanins [21]. However, these forms of colour are unstable and polymerize to create more complex and more stable pigments. The degree of polymerization is mainly affected by the origin of tannin, aging time and aging conditions [15,21,26]. Under the same oenological condition (Figure 2A), the amount of total coloured anthocyanins in control was significantly lower than that in tannin treatments. It is understandable that total anthocyanin content in each tannin treatment is quite similar. Coloured anthocyanin can be increased by high dosage of grape seed tannin (TV), but no significance was observed between oak tannin (RE) and grape skin tannin (TS). The concentration of polymeric anthocyanins in the control is slightly higher than that in the tannin treatments (Figure 2B). This phenomenon is mainly due to the development of acetaldehyde, which can be synthesized by yeast or formed from ethanol by chemical oxidation and react with anthocyanin to promote their polymerization with flavanols [18]. However, other researchers have reported that oenological tannins indirectly inhibited the yeast activity to produce enough acetaldehyde or hydroxycinnamic acid to react with flavanols and then result in insufficient performance on polymerization of anthocyanin [14]. Meanwhile, polymerization of anthocyanins in various tannin treatments are quite different, high oak tannin content lead to lower degree of polymerization, which is an opposite tendency of grape tannin treatments—TV and TS. This could be related to oak tannin have higher pH which result in lower degree of polymerization value [27]. On the other hand, the addition of oenological tannin directly promoted concentration of monomeric anthocyanin, which positively influenced wine colour performance. This was significantly higher than that in the control, particularly for RE, which possessed the highest concentration of monomeric anthocyanin. Hence, it is predicted that these unstable anthocyanins could be involved in a high level of copigmentation to combine with copigments and other molecules, such as flavonoid, phenolic acid and metallic cations during aging process [13,28,29]. However, one point should be noticed that formation of copigmentation complexes is not always a protective factor against anthocyanin disappearance in wines, but depends on the type of copigments and the strength of the interaction established [30].

Anthocyanins are the red pigment in grapes, located mainly in skin and flesh and are made up with five main anthocyanidins: cyanidin, peonidin, delphinidin, petunidin and malvidin, which are dominant in grapes and usually combine with monoglucoside [31,32]. According to the result from Table 2, anthocyanins, D3G, C3G, Pt3G, M3G, Pt3GAc, Pn3GAc, M3GAc, Pt3GCm and Pn3GCm were positively correlated with the addition of oenological tannins. This is due to the precursors of these anthocyanins, which belongs to pyranoanthocyanins, were introduced by addition of oenological tannins. Highly stable pigments, Vitisin A (Vit A), Vitisin A-Ac (Vit A-Ac) and Vitisin B (Vit B), which are derived from acetaldehyde and pyruvic acid, respectively, and improve chromatic characteristic of wine during the aging process [5,26,30], were all reduced by tannin addition. Around 55% Vit A + B significantly decreased in RE treatments while 94% Vit A-Ac dramatically decreased in TS treatments. It is due to part of these copigments were absorbed by yeast cell wall during AF [33]. In addition, hydrolysable tannins can restrict yeast metabolism and therefore production of pyruvic acid and acetaldehyde, that are essential for the production of Vit A and Vit B [27]. Regarding the content of M3G (precursor of Vit A + Vit B), more than twice were increased by tannin addition that could be positive to prevent red colour loss from oxidation during long term aging of red wine. Nevertheless, in order to improve the formation of highly stable pyranoanthocyanins (Vit A + Vit B) in red wine, the artificial addition of 500 mg/L pyruvic acid and 200 mg/L acetaldehyde can, respectively, increase 2.03% Vit A and Vit B 1.35% in total anthocyanin [30].

**Figure 3:**
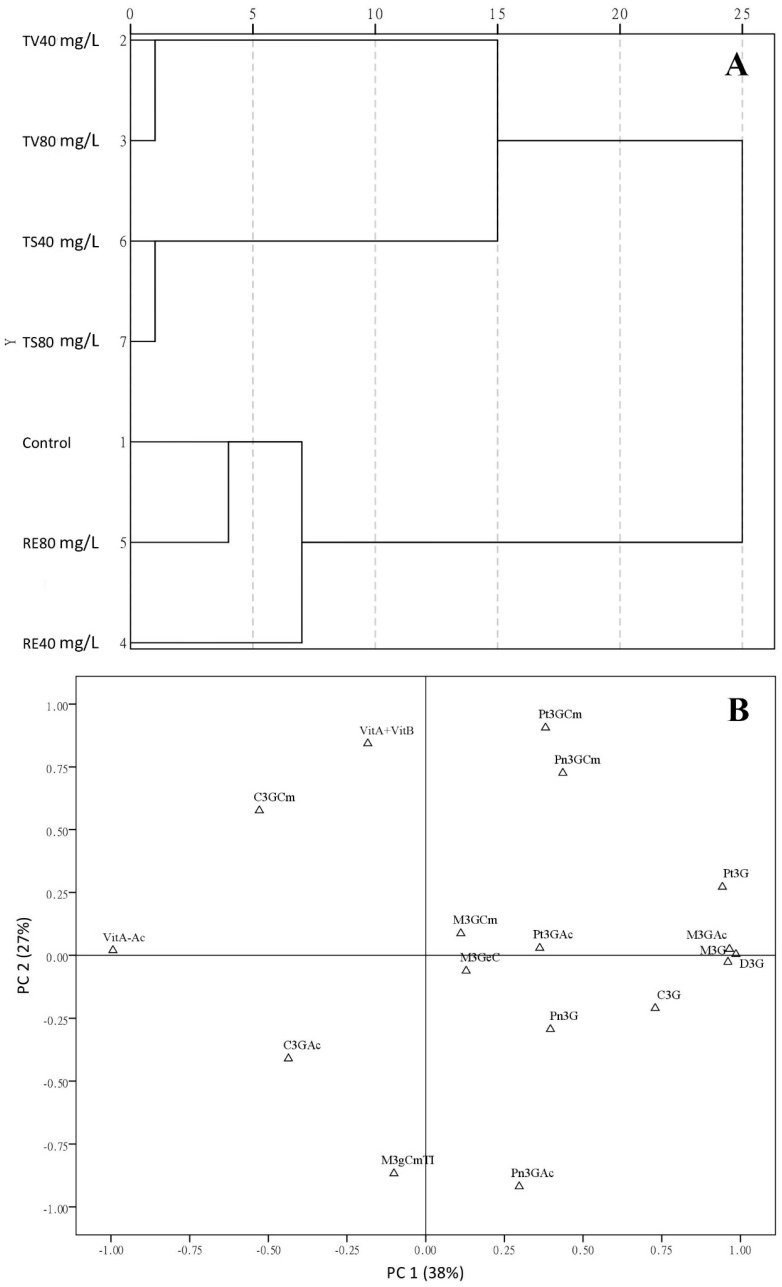
Cluster analysis plot of treatments (**A**); and principal component analysis plot of components (**B**) based on the influence of oenological tannins on wine anthocyanins. The following nomenclature is used: assays fermented with Tanicol Vintage (TV) 40 and 80 mg/L, Robletan Estructura (RE) 40 and 80 mg/L as well as Tan Sutil (TS) 40 and 80 mg/L. The related abbreviations of anthocyanins have been shown on Methods and Materials.

To ensure a comprehensive understanding on anthocyanin evolution, cluster analysis (CA) and principal component analysis (PCA) (Figure 3) were jointly carried out to generate an evaluation of tannin treatments. Cluster analysis (CA) is a multivariate statistical technique used to group elements (or variables) and trying to achieve maximum homogeneity in each group as well as the biggest difference between them. Two main groups were observed at scale 20 on Figure 3A. It is reasonable that the anthocyanins of TV treatment is close to TS treatment since both of these tannins are derived from grape berry (seed and skin). Control was grouped with RE treatment, which derived from French oak (hydrolysable tannin); therefore, the anthocyanin composition is significantly different from that in TV and TS treatments (condensed tannin). The relations between anthocyanins and treatments were reflected by PCA (Figure 3B). Anthocyanins, such as M3GCm, M3GeC, Pt3GAc, Pn3G, C3G, Pt3G, M3GAc, M3G and D3G were all positively related to PC 1, which was dominated by TV and TS treatments. Based on the results of Table 2 and CA (Figure 3A), anthocyanin profile of the fermentations with grape tannins can be confirmed as one group and directly influenced by TV and TS additions. Conversely, Vit A-Ac is located at the end of the negative side of PC 1 since this anthocyanin was affected by TS addition. Vit A + Vit B was placed on the positive side of PC 2, which was statistically related to control and part of RE 80 mg/L. Comparatively speaking, grape seed tannin (TV), contains oligomeric proanthocyanidin which possesses high antioxidant activity to protect the wine from colour loss and benefit to aroma complexity [3,6,34], and showed more effective contributions to pyranoanthocyanins and monomeric anthocyanins than TS and RE treatments in red wine fermentation. In conclusion, the application of TV 40 mg/L had similar effect on anthocyanins to TS treatments, and could be quite promising to improve red wine colour during aging according to its positive anthocyanins evolutions and antioxidant effects.

### 2.4. The Influence of Oenological Tannins on Volatile Compounds

Wine aromas are made up of several hundred volatile compounds, mainly developed from four mechanisms: grape metabolism, biochemical reactions occurring prior to fermentation, metabolism of microorganisms responsible for alcoholic and malolactic fermentation and chemical or enzymatic reactions post fermentation [3,8,34]. The volatile compounds of the seven treatments are shown in Table 3. Tannin treatments significantly reduced acetaldehyde concentration, which was regarded as beneficial compounds for colour stability since it acts as a precursor of Vit B [26,30]. Obviously, the amount of ethyl acetate (pineapple flavour, 7.5 mg/L as threshold) were all increased by oenological tannin addition into acceptable levels in all trials, which were below the limitation of tolerance (150 mg/L) otherwise excessive amount would give undesirable glue odour to olfactory properties [3]. Interestingly, higher alcohols (isobutanol, active amyl alcohol, and isopentanol) were only promoted by TV treatment. On the contrary, RE and TS treatment inhibited the development of higher alcohols from AF. Isoamyl acetate, which could contribute a fruity aroma described as banana, was significantly increased in tannin treatments, where the concentrations were less than 10 mg/L but still outclassed the threshold of 0.26 mg/L [3]. Ethyl lactate production, possessing fruity aroma, was moderately less than threshold 14 mg/L in all experimental trials. Acetoin in tannin treatments was decreased across all trials, although the amount was less than 100 mg/L, which is the threshold to produce an unpleasant buttery flavour [3,16]. Moreover, oenological tannin application can be considered to be favourable for 2-phenylethanol, which is connected with floral aromas and positive for red wine. However, tannin treatment affected methanol and diacetyl development, which were all increased above tolerant limits at 130 mg/L and 5 mg/L respectively, and could result in slight adverse impacts on olfactory properties [4,16].

**Figure 4:**
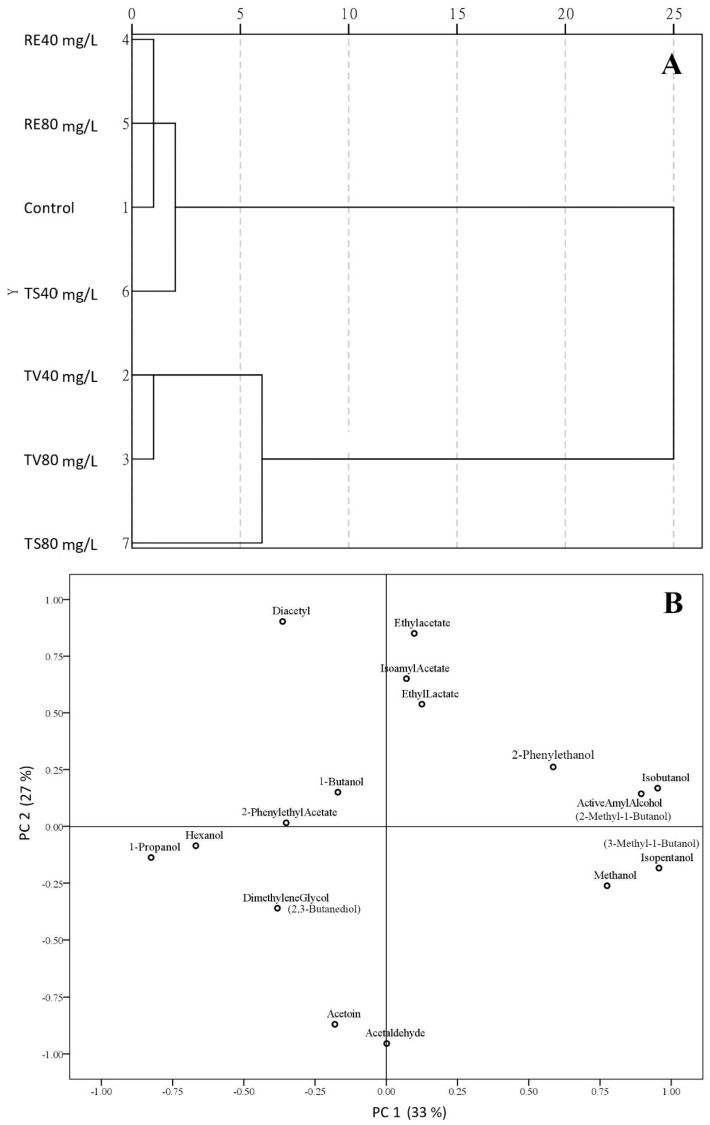
Cluster analysis plot of treatments (**A**); and principal component analysis plot of components (**B**) for the influence of oenological tannins on wine volatile compounds. The following nomenclature is used: assays fermented with Tanicol Vintage (TV) 40 and 80 mg/L, Robletan Estructura (RE) 40 and 80 mg/L as well as Tan Sutil (TS) 40 and 80 mg/L. Meanwhile, Same chemicals with the different name were distinguished on PCA plot: Isoamyl Acetate (3-methyl-1-butanol), Active Amyl Alcohol (2-methyl-1-butanol) and Dimethlene Glycol (2,3-butanediol).

In order to get a more comprehensive understanding of volatile compounds, CA and PCA (Figure 4) were jointly carried out to generate an evaluation of tannin treatments. The effects of seven treatments on volatile compounds were grouped on Figure 4A. With unique standard dimension, scales (0–25) reflected the mapping results of different evaluation indexes (Euclidean distance and cosine, etc.). As a result, the two groups were observed at scale 5, volatile compounds of TV treatment and TS 80 mg/L (tannins derived from grape seed and skin) were regarded as one group according to their high similarity. Meanwhile, volatile compounds of RE treatments (oak tannin) were grouped with control and TS 40 mg/L. On the other hand, principal component analysis was carried out to give an overall description of distributions of 16 volatile compounds [3,25], where PC 1 explained 33% variation cross volatile compounds while PC 2 explained 27% (Figure 4B). The great majority of esters, including ethyl acetate, isoamyl acetate and ethyl lactate, highly contributed to PC 2. Meanwhile, alcohols highly contributed to PC 1, especially for higher alcohols, isobutanol, active amyl alcohol and isopentanol were placed on the positive side of PC 1. Based on the result from Table 2 and CA (Figure 4A), 1-propanol and dimethylene glycol, which were negatively influenced by TV addition, were located on the negative side of PC 1. Moreover, the change of higher alcohols was also strongly correlated with TV treatment, which can be regarded as the main element of PC 1. Diacetyl (positive correlation), acetoin (positive correlation) and acetaldehyde (negative correlation) in RE treatment, which was verified by CA as the main factor of PC 2, can be significantly affected by RE addition. In conclusion, TV treatment is preferred to improve aroma complexity in terms of positive linkage with the evolution of higher alcohols.

### 2.5. The Influence of Oenological Tannins on Sensorial Properties

**Figure 5:**
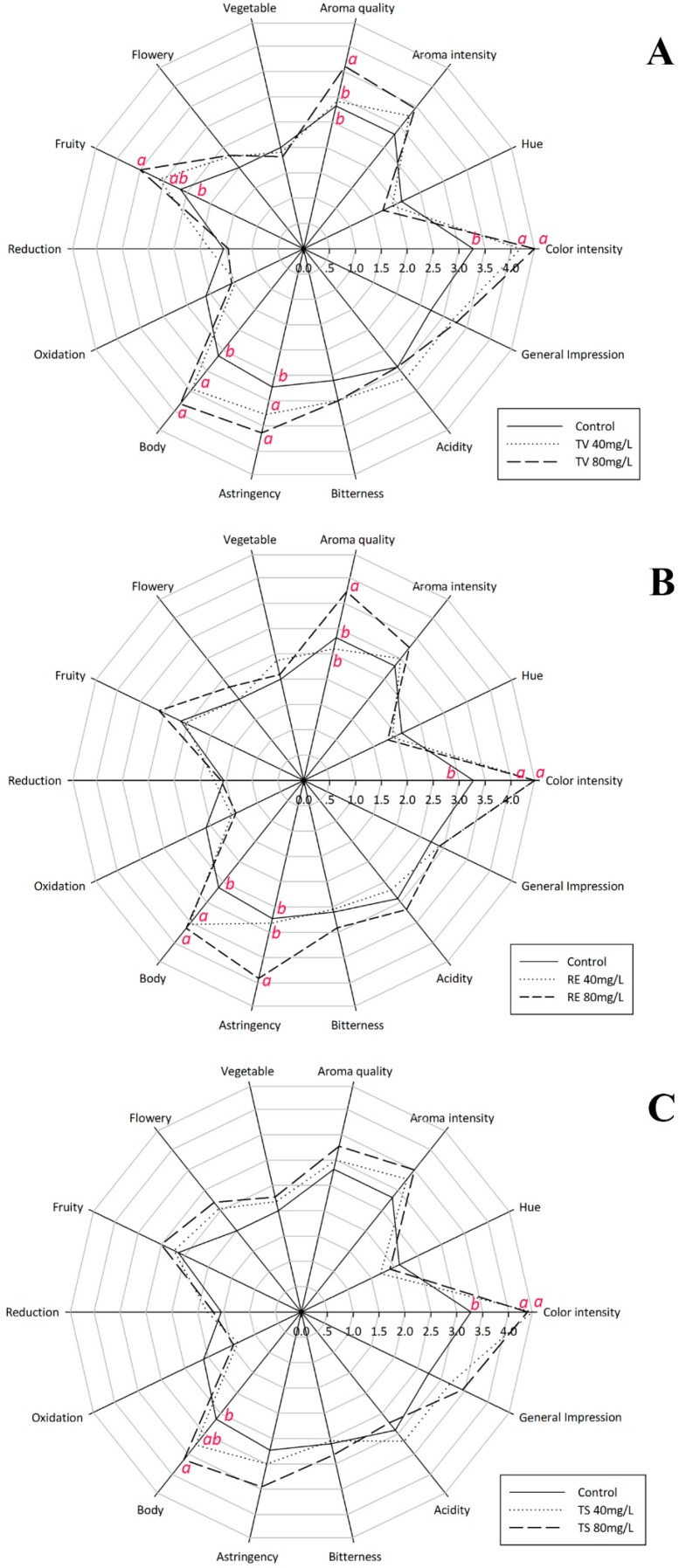
Radar plots, the influence of oenological tannins on sensorial properties. Assays fermented with: Tanicol Vintage (TV) 40 and 80 mg/L (**A**); Robletan Estructura (RE) 40 and 80 mg/L (**B**); and Tan Sutil (TS) 40 and 80 mg/L (**C**). Different letters indicate the significant differences were conducted by Duncan´s new multiple range test at *p* < 0.05.

Sensory influence of oenological tannins has been shown in Figure 5. It is understandable that the oxidation perception of tannin treated wine was reduced because of highly antioxidant effects of oenological tannins, which also affected wine colour [3,6]. Meanwhile, the decrease in hue (A420/A520) was probably due to some exogenous pigments, which mainly contribute to red colour (A520 was promoted), participated in the wine fermentations by addition of oenological tannins. On the contrary, many properties were significantly promoted by addition of grape seed tannin (TV), particularly, fruity, wine body, astringency, colour intensity and aroma quality, which all positively contribute to wine aroma and mouth feeling. It has been credited by Kai Chen et al. (2016) that aroma complexity, fruity and flowery can be improved during ice wine aging process by addition of oligomeric proanthocyanidins [3], which is a main phenolic component of grape seed tannin and positively influence the development of higher alcohols and some esters, such as ethyl acetate, isoamyl acetate and ethyl lactate (Table 3). Although fruity were significantly promoted by TV addition, vegetable aroma showed an opposite result. Moreover, grape skin tannin (TS) has parallel effects with TV treatments on each benchmark. Comparing with the wines treated with TS, TV and RE treatments significantly increased wine astringency that would affect the balance of the young red wine. Gelatin results also indicated the TV and RE treatments increased astringency more than the TS treatment (Table 1).

## 3. Materials and Methods

### 3.1. Microorganism

*Saccharomyces cerevisiae* strain (7VA), which possess the high capability to produce pyruvic acid and acetaldehyde [16], was used in this study. It was selected from “Laboratorio de Tecnología de Alimentos” of E.T.S.I. Agronomos in the Madrid collection [17]. The 7VA suspensions were cultivated at 24 °C for 48 h until initial inoculation scale was controlled approximately as 10^7^ CFU/mL.

### 3.2. Micro-Vinifications

All fermentations were undertaken using must from *Vitis vinifera* Syrah grapes grown at Socuéllamos, Ciudad Real in Castilla la Mancha, Spain. Syrah juice was extracted with classic flash thermovinification from fresh must in order to release more colour and tannins. The process heats grapes to a high temperature (about 82 °C) in a few seconds, then immediately pump the fruit into a vacuum chamber for depressurization. The treatment not only is a fast extraction of colour and tannin without influence on must sensory quality but also sanitized the microbes on berry skins to a certain extent [36]. The juice was carried out with flash pasteurization in an autoclave for 1 min at 105 °C in 24 L tanks for sanitation. Otherwise, spontaneous fermentation would affect final wine components. Sugars were amended up to 230 g/L, the final pH was 3.2, lactic and acetic acids were less than 0.1 g/L. To facilitate the fermentation, nutrients were added at a concentration of 0.4 g/L (Nutrient Vit-Lallemand, Montreal, PQ, Canada). Seven experimental trials were conducted in triplicate: (i) inoculating Syrah must without tannin addition as control; (ii) inoculating Syrah must after being treated with TV 40 mg/L; (iii) inoculating Syrah must after being treated with TV 80 mg/L; (iv) inoculating Syrah must after being treated with RE 40 mg/L; (v) inoculating Syrah must after being treated with RE 80 mg/L; (vi) inoculating Syrah must after being treated with TS 40 mg/L; and (vii) inoculating Syrah must after being treated with TS 80 mg/L. In these seven trials, three types of oenological tannin, TV derived from grape seeds (*Vitis vinifera*), RE derived from toasted French oak (*Querus robur* and *Quercus petraea*) and TS derived from grape skin (*Vitis vinifera*), were all provided by Agrovin S.A. (Ciudad Real, Spain). Each alcoholic fermentation trial was carried out in 150 mL micro vessel caped with a sulphuric acid (Panreac, Barcelona, Spain) filled Müller valve (Alamo, Madrid, Spain) to avoid microbial contamination while releasing CO_2_. All the fermentation trials were accomplished at 25 °C until no weight loss was detected. At the end of the AF, the wines were centrifuged (5000 rpm, 10 min) and then transferred into 125 mL aseptic brown glass bottles, well-sealed and placed at 4 °C for the following analysis.

### 3.3. Analytical Determinations of Physical-Chemical Parameters

Basic fermented parameters, such as alcohol content (13.5%–14% *v*/*v*), residual glucose and fructose (0.3–0.5 g/L), pH (3.–3.2), acetic acid (0.4–0.5 g/L), l-malic acid (3.2–3.6 g/L) and lactic acid (0 g/L) were determined with an Y15 enzymatic autoanalyzer (Biosystems S.A., Barcelona, Spain). These analyses were performed using the appropriate kits from Biosystems enterprise. The Y15 equipment was calibrated with the external standards that are provided in every kit by Biosystems. No significant difference of fermented data was observed in different experimental trials.

### 3.4. Analysis of Phenolic Parameters

An Agilent 8453 UV-Visible spectrophotometer (Santa Clara, CA, USA) was used for the phenolic parameters. Samples were analysed in a quartz cuvette with 1 mm path length and a range of 200 to 1100 nm. Absorbance at 420 nm, 520 nm, and 620 nm was measured and then colour intensity was calculated as the sum of absorbance at the three wavelengths, while hue was calculated as the ratio between the absorbance at 420 nm and 520 nm. Total tannin content was carried out with the acid hydrolysis methods from Ribereau-gayon et al, 1966 [8]. Moreover, five important phenolic parameters were also carried out:

1. Gelatine index, related to the percentage of tannins are able to combine with protein, and mainly used to detect astringent tannins in wine [23].
2. PVPP index, polyvinylpolypyrrolidone is a “protein-like” fining agent with an affinity for low molecule weight phenolics, used for binding with and removing smaller phenolic such as catechins, and evaluate wine astringency and colour [24].
3. HCL index, the percentage of tannins polymerized with polysaccharides and salts [8].
4. Ethanol index, the percentage of tannins that can combine with polysaccharides [8].
5. Vanillin index, expressed with mg/L of catechin, which is unstable carbocations that are converted into red condensation products [8].

### 3.5. Analysis of Wine Anthocyanin Composition and CIELAB Scale

The contribution of the coloured, monomeric and polymeric anthocyanin to total anthocyanin was determined following the method proposed by Boulton [13]. All wine samples were centrifuged 10 min at 5000 rpm, 4 °C before determination. Equations are as follows:

Total anthocyanin (mg/L) = 18.9 × A_520-H_^*^ (1)

Coloured anthocyanin (mg/L) = 18.9 × (A_520_^*^ − A_520-S_^*^) (2)

Polymeric anthocyanin (mg/L) = 18.9 × (5/3)A_520-S_^*^ (3)

Monomeric anthocyanin (mg/L) = 18.9 × [A_520-H_^*^ − (5/3)A_520-S_^*^] (4)

the explanations of relative indexes in above are as follows: A_520_^*^ = A_520_ × 10 (wine was directly detected at wavelength 520 nm with 1 mm cuvette); A_520-S_^*^ = A_520-S_ × 1.05 × 10 (100 μL 6% sodium metabisulphite was added into 2 mL wine sample, vortex blending and then wait 1 min, detection was conducted under 520 nm wavelength with 1 mm cuvette in terms of A_520-S_); and A_520-H_^*^ = A_520-H_ × 100 (100 μL wine sample was added into 10 mL 1M HCL, vortex blending and then wait 4 h at 25 °C, detection was conducted under 520 nm wavelength with 1 cm cuvette in terms of A_520-H_).

In addition, CIELAB scale, L*, a*, b* are a colour scale based on the Opponent-Colour Theory that assumes the receptors in the human eye perceive colour as the following pairs of opposites [37]. L* scale: Light vs. dark where a low number (0–50) indicates dark and a high number (51–100) indicates light. a* scale: Red vs. green where a positive number indicates red and a negative number indicates green. b* scale: Yellow vs. blue where a positive number indicates yellow and a negative number indicates blue [37,38].

### 3.6. Analysis of Volatile Compounds

The concentrations of 17 volatile compounds, acetaldehyde, methanol, 1-propanol, diacetyl, ethyl acetate, isobutanol, 1-butanol, acetoin, active amyl alcohol (2-methyl-1-butanol), isopentanol (3-methyl-1-butanol), ethyl lactate, diethylene glycol (2,3-butanediol), isoamyl acetate, hexanol, 2-phenylethanol, and 2-phenylethyl acetate, all of which can influence on wine aromatic profile, were measured at the end of the alcoholic fermentations by Agilent Technologies 6850 gas chromatograph with a flame ionization detector (Hewlett-Packard, Palo Alto, CA, USA). The apparatus was calibrated with 4-methyl-2-pentanol as an internal standard at 50 mg/L. GC quality standard reagents were purchased from Fluka, Sigma-Aldrich Corp. (Buchs SG, Switzerland). The system was prepared with a DB-624 column (60 m × 250 μm × 1.40 μm), injector temperature was 250 °C and the detector temperature 300 °C. The column temperature was 40 °C for the first 5 min, rising linearly by 10 °C/min until reaching 250 °C and then maintained for 5 min. Hydrogen was used as the carrier gas, which was provided by a hydrogen generator (LNI Schmidlin SA, Geneva, Switzerland). The flow rate was 22.1 L/min, injection split ratio was 1:10, and detection limit was 0.1 mg/ L. One hundred microliters of internal standard (50 mg/L) was added to 1 mL test samples and filtered through syringe membrane filters (0.45 μm pore size) (Teknokroma, Barcelona, Spain). They were then placed in 1.5 mL glass vials sealed with a PTFE/silicon septum. One microliter of this filtrate was injected into the GC apparatus [4,16]. Each wine sample was done in triplicate.

### 3.7. Anthocyanin Analysis by HPLC/ESI-MS

Seventeen kinds of anthocyanins were determined by using an Agilent Technologies (Palo Alto, CA, USA) series 1100 HPLC equipped with a diode array detector and a quadrupole mass spectrometer with an electrospray interface. Gradients of solvent A (water/formic acid, 95:5, *v*/*v*) and B (methanol/formic acid, 95:5, *v*/*v*) were used in a reverse-phase Kinetex C18 column (Phenomenex, Torrance, CA, USA) (100 mm × 4.6 mm; particle size 2.6 μm) as follows: 20%–50% B linear (0.8 mL/min) from 0 to 27 min, 50% B from 27 to 28 min, 20%–50% B linear (0.8 mL/min) from 28 to 29 min, and re-equilibration of the column from 29 to 30 min. Detection was performed by scanning in the 500–600 nm range. Quantification was performed by comparison against an external standard at 525 nm and expressed as a function of the concentration of M3G (Extrasynthèse, Genay, France). The different anthocyanins were identified by their retention times with respect to the majority anthocyanin M3G, and by comparing the UV–visible and mass spectra with data in the literature [15,26,30]. The electrospray ionization variables were: drying gas (N2) flow rate 10 mL/min; temperature 350 °C; nebulizer pressure 380 Pa (55 psi); and capillary voltage 4000 V. Mass spectrometry was performed in positive mode scanning from *m*/*z* 100 to *m*/*z* 1500, using a fragment voltage of 150 V from 0 to 23 min. One hundred microliters sample of previously filtered (0.45 μm membrane filters made of cellulose methylic esters (Teknokroma, Barcelona, Spain)) wines were injected into the HPLC apparatus. The detection limit was 0.1 mg/L. Each wine treatment was done in triplicate.

### 3.8. Sensory Analysis

Sensory analysis was conducted with the method organized by professional panellists from the Polytechnic University of Madrid [4,16]. The experimental wines were evaluated with blind tasting by a team of experienced wine tasters (6 females and 4 males). No specific training was carried out prior to tasting sessions. Previous to the tasting session, the studied parameters were established by consensus. All employees were from the Chemistry and Food Technology Department (UPM, Madrid, Spain). Seven blended wines which were respectively made from the repetition mixture of each treatment, were evaluated in a randomized order once. The wines were presented in clear tasting glasses identified with numbers from one to seven in an air-conditioned (20 °C) tasting room. Fifteen millilitres of each wine was served at 14 °C. The panellists were asked to rate the wines on 14 attributes according to an unstructured scale 0 (absent) to 5 (very intense), to rate the intensity of the parameters. Additionally, the panellists were asked to name descriptors as free comments for each wine sample.

### 3.9. Statistics

All the statistical analysis of experimental data were carried out by using SPSS 19.0 software (IBM SPSS Inc., Chicago, IL, USA). For each tannin-treated wine sample, group differences were identified by ANOVA followed by a post hoc comparison (Tukey’s test at *p* < 0.05). Moreover, cluster analysis, mainly focus on tannin treatments analysis, was performed using Ward´s method and Squared Euclidean distance as distance option. Meanwhile, principal component analysis with the method of varimax rotation, mainly used for various chemical components analysis, was carried out with the aim of highlighting the main contributors of the variance.

## 4. Conclusions

Overall, the data obtained suggested that the pre-fermentative addition of oenological tannins could represent a promising option to improve wine colour, aroma and sensorial properties of young red wine. Especially for the tannins derived from grape seeds (TV), the low dosage is positive and practical to moderately improve wine colour stability and aroma complexity. Further research will focus on mixed culture fermentation with tannins which could influence on wine quality.

## Figures and Tables

**Table 1:** Analysis of phenolic parameters of oenological tannins treated wines after AF.

Phenolic Parameters	Control	TV (40 mg/L)	TV (80 mg/L)	RE (40 mg/L)	RE (80 mg/L)	TS (40 mg/L)	TS (80 mg/L)
Total Tannins (g/L)	2.96 ± 0.10 ^b^	3.11 ± 0.35 ^a^	3.04 ± 0.19 ^b^	3.65 ± 0.29 ^a^	3.08 ± 0.09 ^a,b^	3.03 ± 0.24 ^b^	3.02 ± 0.12 ^b^
pH	3.27 ± 0.03	3.40 ±0.01	3.33 ± 0.02	3.41 ± 0.01	3.35 ± 0.03	3.32 ± 0.01	3.43 ± 0.01
Hue	0.55 ± 0.02 ^a^	0.52 ± 0.02 ^b^	0.51 ± 0.01 ^b^	0.53 ± 0.01 ^a,b^	0.54 ± 0.01 ^a,b^	0.55 ± 0.01 ^a,b^	0.52 ± 0.04 ^a,b^
Colour intensity	2.38 ± 0.05 ^d^	2.77 ± 0.01 ^b^	2.85 ± 0.06 ^a^	2.71 ± 0.02 ^c^	2.79 ± 0.00 ^a,b^	2.76 ± 0.04 ^b,c^	2.80 ± 0.02 ^a,b^
Gelatine index (%)	66.95 ± 4.83 ^b,c^	65.56 ± 4.19 ^b,c^	74.80 ± 1.70 ^a,b^	64.02 ± 12.68 ^b,c^	87.31 ± 7.64 ^a^	75.52 ± 0.48 ^a,b^	54.08 ± 10.68 ^c^
PVPP index (%)	0.67 ± 0.09 ^c^	0.91 ± 0.07 ^a^	0.84 ± 0.06 ^a,b^	0.77 ± 0.13 ^b,c^	0.70 ± 0.04 ^b,c^	0.69 ± 0.03 ^b,c^	0.80 ± 0.13 ^a–c^
HCL index (%)	17.12 ± 6.24 ^c^	48.00 ± 26.29 ^b^	70.66 ± 21.82 ^a,b^	92.36 ± 4.25 ^a^	53.44 ± 26.35 ^b^	93.58 ± 4.68 ^a^	67.90 ± 22.45 ^a,b^
Vanillin Index (g/L of Catechin)	1.03 ± 0.06 ^c^	1.05 ± 0.03 ^c^	1.08 ± 0.04 ^c^	1.31 ± 0.01 ^a^	1.20 ± 0.08 ^b^	1.29 ± 0.09 ^a,b^	1.36 ± 0.05 ^a^
Ethanol index (%)	91.33 ± 5.23 ^d^	47.32 ± 17.13 ^a–c^	27.56 ± 18.08 ^a^	61.85 ± 13.59 ^b,c^	58.73 ± 6.65 ^b,c^	68.13 ± 8.04 ^c^	41.09 ± 13.96 ^a,b^
Total Anthocyanin (mg/L)	516.6 ± 18.36 ^b^	737.1 ± 79.13 ^a^	756.63 ± 37.29 ^a^	731.43 ± 13.23 ^a^	798.84 ± 17.46 ^a^	773.64 ± 11.39 ^a^	760.41 ± 13.93 ^a^

In the same row, different letters indicate the significant differences were conducted by Duncan´s new multiple range test at *p* < 0.05. Meanwhile, TV stands for Tanicol Vintage, RE stands for Robletan Estructura and TS stands for Tan Stutil.

**Table 2:** HPLC-MS results of anthocyanins detected in wine samples fermented by oenological tannin treatments.

Anthocyanins (mg/L)	[M]^+^ *(m/z)*	Fragments *(m/z)*	Control	TV (40 mg/L)	TV (80 mg/L)	RE (40 mg/L)	RE (80 mg/L)	TS (40 mg/L)	TS (80 mg/L)
D3G	465	303	16.58 ± 0.69 ^b^	45.07 ± 2.77 ^a^	48.21 ± 2.38 ^a^	44.86 ± 3.69 ^a^	45.92 ± 1.44 ^a^	46.92 ± 4.62 ^a^	50.79 ± 4.71 ^a^
C3G	449	287	0.20 ± 0.04 ^c^	0.38 ± 0.01 ^b,c^	0.63 ± 0.12 ^b^	1.03 ± 0.12 ^a^	1.19 ± 0.16 ^a^	0.81 ± 0.06 ^a^	1.25 ± 0.08 ^a^
Pt3G	479	317	21.24 ± 2.34 ^c^	40.11 ± 6.04 ^b^	48.48 ± 1.62 ^a^	39.26 ± 1.44 ^b^	40.75 ± 1.21 ^b^	49.55 ± 0.30 ^a^	48.86 ± 4.10 ^a^
Pn3G	463	301	6.88 ± 2.10 ^b,c^	6.99 ± 1.13 ^b,c^	5.07 ± 0.69 ^c^	11.00 ± 0.20 ^a^	11.96 ± 0.46 ^a^	8.38 ± 0.44 ^b^	12.12 ± 0.33 ^a^
M3G	493	331	113.86 ± 4.30 ^b^	238.79 ± 20.62 ^a^	247.79 ± 9.99 ^a^	225.65 ± 10.83 ^a^	228.03 ± 6.02 ^a^	238.82 ± 4.26 ^a^	232.40 ± 13.99 ^a^
Vit A + Vit B	561/517	399/355	7.68 ± 1.70 ^a^	4.28 ± 0.78 ^b^	4.03 ± 0.37 ^b^	3.17 ± 0.17 ^b^	3.74 ± 0.11 ^b^	8.68 ± 0.07 ^a^	8.37 ± 0.68 ^a^
Vit A-Ac	603	399	4.10 ± 0.43 ^a^	0.77 ± 0.09 ^b^	0.73 ± 0.01 ^b,c^	0.66 ± 0.13 ^b,c^	0.61 ± 0.05 ^b,c^	0.38 ± 0.01 ^c,d^	0.15 ± 0.09 ^d^
C3GAc	491	287	0.99 ± 0.59 ^a^	0.93 ± 0.08 ^a^	0.97 ± 0.03 ^a^	0.92 ± 0.055 ^a^	0.85 ± 0.03 ^a^	0.36 ± 0.07 ^b^	0.86 ± 0.20 ^a^
Pt3GAc	521	317	2.38 ± 0.20 ^e^	8.25 ± 0.67 ^a^	8.16 ± 0.57 ^a^	5.43 ± 0.29 ^c^	3.31 ± 0.46 ^d^	2.39 ± 0.02 ^e^	6.72 ± 0.54 ^b^
M3GeC	809		3.33 ± 1.07 ^b^	5.21 ± 1.42 ^a^	6.28 ± 0.86 ^a^	3.25 ± 0.95 ^b^	4.70 ± 0.76 ^b^	6.38 ± 0.77 ^a^	0.43 ± 0.05 ^c^
Pn3GAc	505	301	3.76 ± 0.02 ^e^	7.69 ± 0.65 ^c^	7.69 ± 0.25 ^c^	24.43 ± 1.06 ^a^	16.54 ± 1.33 ^b^	5.72 ± 0.49 ^d^	5.36 ± 0.30 ^d^
M3GAc	535	331	26.60 ± 1.65 ^c^	47.20 ± 3.43 ^b^	50.29 ± 1.64 ^b^	52.22 ± 5.28 ^b^	58.924 ± 1.50 ^a^	61.54 ± 1.48 ^a^	58.78 ± 5.38 ^a^
Pt3GCm	625	317	1.61 ± 0.18 ^c^	3.55 ± 1.28 ^b^	4.17 ± 0.29 ^b^	ND	ND	7.93 ± 0.33 ^a^	6.99 ± 0.93 ^a^
Pn3GCm	609	301	ND	0.12 ± 0.01 ^b^	0.13 ± 0.01 ^b^	ND	ND	37.82 ± 0.61 ^a^	36.033 ± 4.50 ^a^
M3GCm	639	331	ND	25.99 ± 2.56	28.68 ± 2.03	ND	ND	ND	ND
C3GCm	595	287	0.85 ± 0.21 ^a^	ND	ND	ND	ND	0.23 ± 0.01 ^b^	0.80 ± 0.09 ^a^
M3GCmTI	639	331	12.81 ± 0.30 ^b^	ND	ND	44.86 ± 3.69 ^a^	18.94 ± 2.48 ^b^	ND	ND

In the same row, different letters indicate the significant differences were conducted by Duncan´s new multiple range test at *p* < 0.05. Meanwhile, TV stands for Tanicol Vintage, RE stands for Robletan Estructura and TS stands for Tan Stutil. The values of [M]^+^ (*m*/*z*) and Fragments (*m*/*z*) were referenced by following literatures: Antonio Morata et al., 2007 and Antonio Morata et al., 2012 [26,30].

**Table 3:** The influence of oenological tannins on development of volatile compounds.

Volatile Compounds (mg/L)	LRI ^§^	Control	TV (40 mg/L)	TV (80 mg/L)	RE (40 mg/L)	RE (80 mg/L)	TS (40 mg/L)	TS (80 mg/L)
Acetaldehyde	800	10.97 ± 2.65 ^a^	7.99 ± 0.55 ^c^	8.37 ± 0.10 ^c^	10.56 ± 0.32 ^b^	9.94 ± 0.39 ^b,c^	11.54 ± 0.77 ^b^	11.61 ± 0.41 ^b^
Methanol	879	139.10 ± 9.27 ^a,b^	142.53 ± 7.44 ^a^	137.06 ± 3.85 ^a,b^	132.98 ± 3.15 ^a,b^	129.96 ± 2.37 ^b^	129.81 ± 3.10 ^b^	130.18 ± 2.63 ^b^
1-Propanol	1069	37.66 ± 2.07 ^a^	30.63 ± 2.36 ^b^	28.32 ± 0.55 ^b^	39.54 ± 1.46 ^a^	36.89 ± 0.67 ^a^	40.55 ± 3.87 ^a^	40.62 ± 0.57 ^a^
Diacetyl	1585	2.00 ± 0.35 ^e^	7.30 ± 0.38 ^d^	7.63 ± 0.24 ^d^	8.77 ± 0.34 ^c^	8.79 ± 0.18 ^c^	9.60 ± 0.43 ^b^	10.40 ± 0.30 ^a^
Ethyl acetate	834	41.87 ± 3.06 ^b^	54.43 ± 4.46 ^a^	53.38 ± 3.28 ^a^	49.41 ± 1.41 ^a^	52.31 ± 1.60 ^a^	53.67 ± 0.69 ^a^	54.83 ± 4.73 ^a^
Isobutanol	1098	14.27 ± 1.59 ^b^	19.58 ± 0.71 ^a^	19.95 ± 1.15 ^a^	13.63 ± 0.28 ^b,c^	13.32 ± 0.25 ^b,c^	13.19 ± 0.24 ^b,c^	12.69 ± 0.19 ^c^
1-Butanol	1173	6.57 ± 2.22	6.35 ± 2.07	7.76 ± 0.55	6.18 ± 1.91	14.34 ± 12.38	7.40 ± 0.30	7.29 ± 0.30
Acetoin	1291	11.17 ± 1.44 ^a^	8.41 ± 0.45 ^b^	8.58 ± 0.08 ^b^	8.80 ± 0.01 ^b^	8.67 ± 0.21 ^b^	8.99 ± 0.15 ^b^	9.10 ± 0.15 ^b^
Active Amyl Alcohol (2-Methyl-1-Butanol)	1086	120.73 ± 31.38 ^b^	159.60 ± 5.48 ^a^	156.78 ± 5.83 ^a^	119.59 ± 1.60 ^b^	118.56 ± 1.62 ^b^	116.08 ± 1.04 ^b^	114.14 ± 3.25 ^b^
Isopentanol (3-Methyl-1-Butanol)	1208	37.51 ± 5.02 ^b^	42.42 ± 1.78 ^a^	42.98 ± 2.31 ^a^	28.47 ± 1.19 ^c^	28.57 ± 1.09 ^c^	28.36 ± 0.61 ^c^	26.34 ± 2.61 ^c^
Ethyl lactate	1363	6.17 ± 0.22 ^b^	6.94 ± 0.65 ^a,b^	8.22 ± 1.47 ^a^	6.61 ± 0.24 ^a,b^	7.07 ± 0.59 ^a,b^	7.65 ± 1.97 ^a,b^	7.11 ± 0.27 ^a,b^
Dimethylene Glycol (2,3-Butanediol)	1692	367.68 ± 38.62 ^a^	273.05 ± 53.77 ^b^	299.53 ± 6.71 ^b^	382.80 ± 19.68 ^a^	370.92 ± 6.43 ^a^	402.08 ± 22.64 ^a^	241.52 ± 7.36 ^c^
Isoamyl acetate	1123	6.79 ± 1.20 ^b^	9.14 ± 1.44 ^a,b^	8.99 ± 1.16 ^a,b^	9.46 ± 0.41 ^a^	8.72 ± 1.90 ^a,b^	8.19 ± 1.55 ^a,b^	9.34 ± 0.43 ^a^
Hexanol	1366	3.83 ± 0.17 ^a^	1.26 ± 2.19 ^b^	2.53 ± 2.19 ^a^	3.92 ± 0.33 ^a^	4.01 ± 0.27 ^a^	3.83 ± 0.06 ^a^	4.05 ± 0.12 ^a^
2-Phenylethanol	1959	38.50 ± 8.81 ^b^	50.68 ± 6.54 ^a,b^	53.89 ± 9.58 ^a^	47.37 ± 2.15 ^a,b^	41.13 ± 7.74 ^a,b^	38.23 ± 8.33 ^b^	38.24 ± 7.78 ^b^
2-Phenylethyl acetate	1850	6.18 ± 0.21	5.82 ± 0.33	5.92 ± 0.44	6.66 ± 1.03	5.98 ± 0.61	6.16 ± 0.55	6.93 ± 1.00

In the same row, different letters indicate the significant differences were conducted by Duncan´s new multiple range test at *p* < 0.05. Meanwhile, TV stands for Tanicol Vintage, RE stands for Robletan Estructura and TS stands for Tan Stutil. ^§^ LRI (linear retention indices) in DB-wax column, which mainly referenced by following literatures: Kai Chen et al., 2016 and Whasley F. Duarte et al., 2010 [3,35].

## Abbreviations

The following abbreviations are used in this manuscript:

AF: alcoholic fermentation
C3G: cyanidin-3-*O*-glucoside
C3GAc: cyanidin-3-*O*-(6′′-acetylglucoside)
C3GCm: cyanidin-3-*O*-(6′′-p coumaroylglucoside)
CA: cluster analysis
D3G: delphinidin-3-*O*-glucoside
M3G: malvidin-3-*O*-glucoside
M3GAc: malvidin-3-*O*-(6′′-acetylglucoside)
M3GCm: malvidin-3-*O*-(6′′-*p*-coumaroylglucoside)
M3GCmTI: malvidin-3-*O*-(6''-*p*-coumaroylglucoside)-*trans* isomer
M3GeC: malvidin-3-*O*-glucoside-ethyl-(*epi*)catechin
PCA: principal component analysis
Pn3G: peonidin-3-*O*-glucoside
Pn3GAc: peonidin-3-*O*-(6′′-acetylglucoside)
Pn3GCm: peonidin-3-*O*-(6′′-pcoumaroylglucoside)
Pt3G: petunidin-3-*O*-glucoside
Pt3GAc: petunidin-3-*O*-(6′′-acetylglucoside)
Pt3GCm: petunidin-3-O-(6′′-pcoumaroylglucoside)
PVPP: polyvinylpolypyrrolidone
RE: Robletan Estructura
TS: Tan Sutil
TV: Tanicol Vintage
Vit A + Vit B: Vitisin A + Vitisin B
Vit A-Ac: Vitisin A-(6′′ acetylglucoside)
